# Dataset of the adapted COVID stress scales for healthcare professionals of the northeast region of Mexico

**DOI:** 10.1016/j.dib.2021.106733

**Published:** 2021-01-09

**Authors:** Gerardo R. Padilla-Rivas, Juan Luis Delgado-Gallegos, Rene de Jesús Montemayor-Garza, Héctor Franco-Villareal, María De los Ángeles Cosio-León, Gener Avilés-Rodriguez, Erika Zuñiga-Violante, Gerardo Salvador Romo-Cardenas, Jose Francisco Islas

**Affiliations:** a Departamento de Bioquímica y Medicina Molecular, Facultad de Medicina, Universidad Autónoma de Nuevo León, Avenida Dr. Eduardo Aguirre Pequeño, Col. Mitras Centro, Monterrey, NL, México; b Instituto de Salud para el Bienestar, Clínica psiquiátrica Dr Everardo Neumann Peña, Carr Matehuala 8, Fracción los Olivos, 78430 Soledad de Graciano Sánchez, SLP, México; c Althian Clinical Research, Calle Capitán Aguilar Sur 669, Col. Obispado Monterrey, NL, México; d Universidad Politécnica de Pachuca, Zempoala, Hidalgo, México; e Facultad de Ingeniería, Arquitectura y Diseño, Universidad Autónoma de Baja California, Ensenada, México; f Universidad de Montemorelos, *Av*. Libertad 1300 Pte, Matamoros 67515, México

**Keywords:** Mexico COVID stress scales, COVID survey, Healthcare professionals stress

## Abstract

The dataset presented examines the levels of stress persisting in healthcare professionals of the Northeast region of Mexico. Using an online platform to obtain data, a survey was developed and distributed through electronic means during a 6-week period covering July and August 2020, considered one of the periods with the highest reported COVID cases in Mexico. Our survey looked at six major stress developing areas: danger, fear of contamination, social economic consequences, xenophobia, compulsive checking and reassurance seeking, and traumatic stress; we added an extra question to assess fear of being an asymptomatic patient. The data was statistically analyzed looking for correlations and dependencies. Thus, helping in policy and decision-making processes to assist and manage stress in healthcare professionals.

**Specifications Table**

**Table:** 

Subject	Public Health and Health policy
Specific subject area	Infection disease and virology
Type of data	Primary data, Dataset
How data were acquired	Data was collected using an online survey written in MS FORMS (Windows ®). The questionnaire is provided as a supplementary file.
Data format	Raw and recategorized/processed
Parameters for data collection	The questionnaire was applied during a 6-week period spanning from July to August 2020, during which information was obtained for a total 112 respondents.
Description of data collection	The questionnaire was conducted through online survey, which was distributed to regional healthcare professionals, via social media such as medical professional social groups on Whatsapp, direct emails and word of mouth from respondents.
Data source location	Country: Mexico Cities: Matamoros Tamps, Nuevo Laredo Tamps, Monterrey NL, and San Luis SLP.
Data accessibility	Database is uploaded on Kaggle repository [1] Direct URL to data:10.34740/KAGGLE/DSV/1731818
Related research article	J.L Delgado-Gallegos, R.J. Montemayor-Garza, G.R. Padilla-Rivas, H. Franco-Villareal and J.F Islas. Prevalence of Stress in Healthcare Professionals during the COVID-19 Pandemic in Northeast Mexico: A Remote, Fast Survey Evaluation, Using an Adapted COVID-19 Stress Scales. Int. J. Environ. Res. Public Health 2020, 17, 7624.

## Value of the Data

• The data is important, as it is one of the initial surveys testing the levels of stress in healthcare professionals in Mexico. The survey is adapted from the COVID Stress Scales (CSS) [2] which looks at the six major psychometric areas involved in anxiety and stress: danger, fear of contamination, social economic consequences, xenophobia, compulsive checking and reassurance seeking, and traumatic stress.
• The data is important as it correlate with current studies which measure the importance of manageable levels of stress within the healthcare community.
• Our results are important as they can help set precedence in the decision making and policy-making processes of how and who should be attending COVID patients, and how security measures should be in place.
• The data was taken during one of the highest peaks of daily reported COVID cases.
• The data looks at one of the major industrial regions in Mexico, because of the industrialized area, the cities involved have high levels of national and international migration.
• The regions in Mexico where the data was taken has high economic ties to the US.

## 1 Data Description

The dataset provided relates to the adapted COVID Stress Scales (CSS) for healthcare professionals [3] and is available at https://www.kaggle.com/chepox/css-mexico. There are 2 datasets involved: raw and re-categorized/processed data. The RAW.CSV file contains the results for the original questionnaire. All sections used a Likert format to evaluate answers. Sections 1–4 contain 6 questions each, with the exception of section 1, which includes an extra question, evaluating the fear of being an asymptomatic patient or FOBAP. The sections were evaluated as never, little, moderate, much, and extreme. Sections 5–6, also containing 6 questions, were evaluated as never, rarely, sometimes, occasionally, and almost always. The 2 variation of the answers in the Likert format are in accordance to the original format [2].

The second dataset, CSS Mexico database.CSV file, contains re-categorized answers to a set of determined values. On sections 1–4 values were given as followed never=0, little=1, moderate=2, much=3, and extreme=4. Meanwhile, the values given to sections 5–6 were never=0, rarely=1, sometimes=2, occasionally=3, and almost always=4. After assigning values to all answers, we recollected their sum in the columns of Total points per section. Furthermore, we added sections 1 and 4 as a single area (Danger and Contamination), the rest of the sections were subsequently re-branded as areas, and are all found in Total points per area.

Both files contain identifier numbers (ID#) assigned in accordance to participation order, additional questions related to the willingness to take part in the study, levels of professional training/type of training, area of work (RAW.CSV file open answer), and whether they see COVID patients and the number of patients with COVID seen daily. In the CSS Mexico database.CSV file, the information provided in the open answer question referring to the area of work, was re-assigned into the following categories: front line, pediatrics, radiology, ICU, Internal medicine, COVID designated area, surgical, ER, OBGYN, and others. As these categories best represent the overall work area. The overall structure of dataset files is described on Table 1.

**Table 1:** General description of the database files. RAW.CSV contains columns A to AQ. CSS Mexico database.CSV contains columns A to BF.

Columns	Description (translated)
A	ID #
B	Do you wish to participate in the study?
C	What is your profession?
D	What area do you work in?
E	Do you work with patients with coronavirus disease?
F	How many patients do you attend per day?
G to L	Section 1 (Danger)*
M to R	Section 2 (Socioeconomical)*
S to X	Section 3 (Xenophobia)*
Y to AD	Section 4 (Fear of Contamination)*
AE to AJ	Section 5 (Traumatic stress)*
AK to AQ	Section 6 (Compulsive checking and reassurance)*
AS to AY	Total points per section for each individual participant
BA to BF	Total sum per area for each individual participant **

*Individual questions in supplemental data.

**Section 1 and 4 were added together, all other areas have the same values as their according section.

Supplementary data contains the translated version of the modified CSS, as originally applied. The structure of the questionnaire is: initial questions which relate area of work, training and work with patients, next sections 1–6 and finally questions which include if the volunteer has had a previous diagnosis of COVID-19 and if the participant wishes to take part in future studies. The original questionnaire (in Spanish) is available at: https://forms.office.com/Pages/ResponsePage.aspx?id=EZDKymp73kSGHwlaLKiDtwzGchlbZ9ZOiuP9lnTCtMdUOFRMSFhCQkZEVUJCRDBZTlc0UU9HWTJUUi4u.

## 2 Experimental Design, Materials and Methods

We based our study on the application of the adapted CSS. We began by regionalizing the original CSS to the Spanish-speaking healthcare professional community in Mexico [3]. We based the design on the original 36-item questionnaire CSS which is used to measure stress levels and anxiety symptoms in daily life [2]. Our adapted questionnaire for healthcare professionals analyzes six psychometric areas of the CSS: danger and contamination fears (evaluated together as area 1), fears about economic consequences (area 2), xenophobia (area 3), compulsive checking and reassurance seeking (area 4), and traumatic stress symptoms (area 5), and how these all related to the stress of COVID-19 in different areas of the medical field. We next distributed the test online using direct distribution such as professional social media groups, direct email, and WhatsApp to healthcare professionals from the northeast part of Mexico. The targeted population were from Monterrey, San Luis Potosi, Nuevo Laredo, and Matamoros. We applied the questionnaire anonymously, acquiring no personal data, through a 6-week period from July to August 2020. As this time frame represents an interval in which Mexico had the highest peak of daily cases, according to the Mexico health ministry, at the time [4], [5].

We further applied statistical analysis and correlations to the recorded data using IBM SPSS Statistics for Windows, version 23.0 (IBM Corp., Armonk, NY, USA). These tests included Pearson's chi-squared (*p* < 0.05), calculation of degrees of freedom, verisimilitude and linear association. Results from these tests help evaluate the prevalence in the alteration of mental health in healthcare professionals attending COVID-19 patients in Mexico.

In order to define our intervals of evaluation, we devised a scaling system using the following formula EQ1:

(1) $$\text{Interval}\;\text{width}\;=\;\frac{\#\;\text{of}\;\text{categories}-1}{\#\;\text{of}\;\text{results}}$$

There were 5 categories (0, 1, 2, 3, 4) and 4 different results (absent, mild, moderate, and severe) with 6 questions per section, as seen in our manuscript supplemental Table 8 [3]. In order to define the category per each section, we added the total number of points. Table 2 shows the Scoring system that was applied per each section. We should note that, in the particular case of areas, we took together sections 1 and 4 (area of Danger and Contamination), we summed the 2 sections together and doubled the necessary points of Table 2 to maintain consistency when changing category. In all other areas, point system remained the same as in sections. Finally, we calculated a general classification as the sum of all the areas (supplemental Table 8).

**Table 2:** Scoring scale system by total points.

Total of possible points per section*	
Score	Category
0–6	Absent
7–12	Mild
13–18	Moderate
19–24	Severe

*Category changes were determined in relation of score points to # of questions (6 per section).

In addition, we added a question to measure “the fear of being an asymptomatic patient” (FOBAP) and scored it independently (supplemental Table 7). Other items regarding the healthcare professionals, corresponding to the level or type of training, specialties, areas of work, number of COVID-19 patients attended per day, and if the subjects had themselves a previous diagnose of COVID-19, and their willingness to continue participating in follow-up questionnaires, totaling 45 items.

## Ethical Statement

The study was conducted in accordance with the Declaration of Helsinki, and the protocol was approved by the Ethics Committee of Hospital La Misión, Monterrey NL. México. Protocol #PSY-CSS-ESP-001. Participant consent was taken by electronic form. Before opting to partake in the study, the survey informed the participants about the nature of the study.

## CRediT Author Statement

**Gerardo R. Padilla-Rivas:** Formal analysis, Methodology, Writing - original draft; **Juan Luis Delgado-Gallegos:** Conceptualization, Project administration, Writing - original draft; **Rene de Jesús Montemayor-Garza:** Methodology, Validation; **Héctor Franco-Villareal:** Resources, Project administration; **María De los Ángeles Cosio-León:** Visualization; **Gener Avilés-Rodriguez:** Investigation; **Erika Zuñiga-Violante:** Investigation; **Gerardo Salvador Romo-Cardenas:** Validation, Writing - original draft; **Jose Francisco Islas:** Project administration, Supervision, Writing - review & editing.

## Declaration of Competing Interest

All authors declare that they have no competing financial or personal interests.

## Appendix Supplementary materials

Image, application 1

## Footnotes

Supplementary material associated with this article can be found in the online version at doi:10.1016/j.dib.2021.106733.
