# Supplementary material for: Dataset of the adapted COVID stress scales for healthcare professionals of the northeast region of Mexico
Source: Data Brief. 2021 Jan 9;34:106733. doi: 10.1016/j.dib.2021.106733 (PMC7835532; doi:10.1016/j.dib.2021.106733)
Supplement: Supplementary file 1 [file mmc1.docx]

**COVID Stress Scales for healthcare professionals** (*Delgado-Gallegos et al., 2020)*

**Initial Questions:**

1. Do you wish to participate in the study?
2. What is your profession?
3. What area do you work in?
4. Do you work with patients with coronavirus disease?
5. How many patients do you attend per day?

**Section 1 (Danger):**

1. I am worried about catching the virus
2. I am worried of having the virus and being asymptomatic*
3. I am worried that basic hygiene (e.g., handwashing) is not enough to keep me safe from the virus
4. I am worried that our healthcare system is unable to keep me safe from the virus
5. I am worried that I can’t keep my family safe from the virus
6. I am worried that our healthcare system won’t be able to protect my loved ones
7. I am worried that social distancing is not enough to keep me safe from the virus

**Section 2 (Socioeconomical):**

1. I am worried about grocery stores running out of food
2. I am worried about grocery stores running out of cold or flu remedies
3. I am worried about pharmacies running out of prescription medicines
4. I am worried about grocery stores running out of water
5. I am worried about grocery stores running out of cleaning or disinfectant supplies
6. I am worried that grocery stores will close down

**Section 3 (Xenophobia):**

1. I am worried that foreigners are spreading the virus.
2. I am worried that people living out of the the state, might have the virus
3. I am worried about coming into contact with foreigners because they might have the virus
4. I am worried that foreigners are spreading the virus because they’re not as clean as we are
5. If I went to a restaurant that specialized in foreign foods, I’d be worried about catching the virus
6. If I was in an elevator with a group of foreigners, I’d be worried that they’re infected with the virus

**Section 4 (Fear of Contamination):**

1. I am worried that people around me will infect me with the virus
2. I am worried that if I touched something in a public space (e.g., handrail, door handle), I would catch the virus
3. I am worried that if someone coughed or sneezed near me, I would catch the virus
4. I am worried that I might catch the virus from handling money or using a debit machine
5. I am worried about taking change in cash transactions
6. I am worried that my mail has been contaminated by mail handlers

**Section 5 (Traumatic stress):**

| 1. I had trouble sleeping because I worried about the virus |
| --- |
| 1. I had bad dreams about the virus |
| 1. I thought about the virus when I didn’t mean to |
| 1. Disturbing mental images about the virus popped into my mind against my will |
| 1. I had trouble concentrating because I kept thinking about the virus |
| 1. Reminders of the virus caused me to have physical reactions, such as sweating or a pounding heart |

**Section 6 (Compulsive checking):**

1. Checked social media posts concerning COVID-19
2. Checked YouTube videos about COVID-19
3. Sought reassurance from friends or family about COVID-19
4. Checked your own body for signs of infection (e.g., taking your temperature)
5. Asked health professionals (e.g., doctors or pharmacists) for advice about COVID-19
6. Searched the Internet for treatments for COVID-19

**Final questions for future follow-up:**

1. Have you been diagnosed with COVID-19?
2. Are you interested in participating in future mental health questionnaire follow-up?
3. We appreciate your interest, please leave an e-mail.

Fear of being an asymptomatic patient (FOBAP)*
